# Don’t just do it—Conducting and publishing improvement science in infection prevention and antibiotic stewardship

**DOI:** 10.1017/ash.2021.259

**Published:** 2022-03-02

**Authors:** W. Matthew Linam, Kavita K. Trivedi, Joshua K. Schaffzin

**Affiliations:** 1 Department of Pediatrics, Emory University School of Medicine and Children’s Healthcare of Atlanta, Atlanta, Georgia; 2 Division of Communicable Disease Control and Prevention, Alameda County Public Health Department, San Leandro, California; 3 Division of Infectious Diseases, Cincinnati Children’s Hospital Medical Center, Cincinnati, Ohio; 4 Department of Pediatrics, University of Cincinnati College of Medicine, Cincinnati, Ohio

The goal of infection prevention and control (IPC) and antimicrobial stewardship programs is to prevent harm to patients and others from healthcare-acquired infection (HAI) and infection with multidrug-resistant organisms (MDROs). The harm caused by HAIs and MDROs is well documented, with cost estimates upward of $10 billion and deaths upward of 100,000 annually.^1,2^ External pressures, such as public reporting requirements, national ranking lists, and nonpayment for HAIs all carry an expectation of institutions to reduce HAI rates and slow the spread of MDROs.^3–5^ Similarly, value-based models of care expect healthcare systems to demonstrate improvement as evidence of care quality. However, many of these pressures have not yielded the desired outcome, and we remain far from goal achievement.^6,7^ One potential solution, improvement science (also known as implementation science or quality improvement) is a method growing in popularity for its ability to affect outcomes relatively quickly by influencing a system rather than targeting a single intervention or people.^8^ Improvement science is not a simple ‘let’s do something’ approach, and is not meant to bypass rigorous investigation and evaluation. Rather, improvement science possesses methodologies and principles that when followed can generate reproducible results that affect outcomes and enhance a broad range of practice. In this review, we intend to introduce the reader to the discipline of improvement science, providing examples of successful improvement science work in infection prevention and control (IPC) and antimicrobial stewardship in the United States and globally. It is our hope that *Antimicrobial Stewardship and Healthcare Epidemiology* (ASHE) will serve as a platform to report such work in the future, advancing knowledge and practice for the fields of IPC and antimicrobial stewardship.

## Improvement science as a discipline

Improvement science is based on the belief that learning from one another in real time is important and an effective means to achieve goals. Solutions for Patient Safety, an engagement network of >145 North American children’s hospitals, has used this concept of ‘All Teach, All Learn’ to reduce harm to hospitalized children, including HAIs like catheter-associated bloodstream infections (CLABSIs), surgical site infections (SSIs), and catheter-associated urinary tract infections (CAUTIs).^9–11^ Shared learning includes both successes and failures. A key tenet of improvement science is that failing is inevitable and acceptable and can lead to insights that contribute to success. This idea contrasts with more traditional research, in which negative results are omitted from reports or are not published at all.

Similarly, collaborative improvement science efforts have transformed adult care. The Comprehensive Unit-based Safety Program (CUSP) demonstrated the efficacy of care bundles to prevent CLABSIs, which have now become the standard of care.^12^ The importance of provider hand hygiene was first demonstrated by Schemmelweiss >170 years ago (and 14 years before germ theory was introduced), but investigators like Didier Pittet and others conducted the seminal work that led to the World Health Organization (WHO) Five Moments of Hand Hygiene and demonstrated how to measure and improve hand hygiene to impact HAI rates.^13–15^

Improvement science has been used to implement published evidence, shorten the estimated 17-year gap between research publication and adoption, and generate evidence where none exists.^16,17^ In these scenarios, adherence to improvement science methodology and purposeful design and interpretation of planned experimentation are essential to success. Methodologies developed in production industries have been applied in healthcare settings, and numerous frameworks and approaches have been published and publicized through organizations like the Institute for Healthcare Improvement (IHI).^18^ Institutions have developed internal educational collaboratives, some of which are open to nonemployees, to teach and disseminate improvement science.^19^

Common to all methodologies is the requirement for planned experimentation and improvement driven by data. The Model for Improvement, based on the work of W.E. Deming and a core component of the IHI approach, begins with a seemingly simple question: What are we trying to accomplish?^20,21^ It is essential to define scope and goals to explain why improvement is needed at a project’s outset. This emphasis is similar to the ‘What is the problem we are trying to solve?’ question that is an essential part of most basic or clinical research grant proposals. The method then provides a framework for (1) application of improvement fundamentals, including developing a change that will affect improvement (hypothesis or theory); (2) having a feedback mechanism to detect improvement (assay or measure); (3) testing a change before implementing (experimentation); and (4) knowing when to implement the change permanently (conclusion).^22^ A robust analysis methodology, statistical process control (SPC), is used to measure processes and outcomes to detect the true change (known as a ‘special cause’) in a system.^23,24^ Interestingly, some seminal SPC papers were published in *Infection Control and Hospital Epidemiology* by Benneyan in 1998, foreshadowing the partnership between IPC, antimicrobial stewardship, and improvement science.^25,26^

A standard framework for publishing improvement science work already exists, termed the Standards for QUality Improvement Reporting Excellence (SQUIRE) guidelines. Initially proposed in 2005 and published in 2009, the guidelines have undergone modification, most recently in 2016, and they are available online along with explanations and resources to aid in their effective use.^27–30^ The guidelines include 18 items for authors to consider and address when publishing “reports that describe system level work to improve the quality, safety, and value of healthcare, and [use] methods to establish that observed outcomes were due to the intervention(s).”^30^ Authors are encouraged to adapt the guidelines and to exclude items irrelevant to their story to disseminate the knowledge gained in their work. Many journals, including *Antimicrobial Stewardship and Hospital Epidemiology*, require or encourage use of SQUIRE guidelines for submissions of improvement science manuscripts for publication, similar to the standard formats expected for other study types.

## Improvement science and infection prevention

### Environmental cleaning

A key element of HAI reduction efforts over the past 20 years has been improving and ensuring environmental cleaning practices. Florence Nightingale’s demonstration of the importance of cleanliness within healthcare settings is >170 years old, but like hand hygiene, systematic efforts to improve hospital cleaning practices have been implemented more recently.^31^ Studies evaluating the effectiveness of cleaning of high-touch surfaces after patient discharge have shown that only ∼50% of surfaces were cleaned.^32,33^ Application of improvement science principles has improved the consistency of cleaning of hospital-room surfaces and the efficiency of ultraviolet light disinfection.^32,34–36^

### Hand Hygiene

The hand hygiene practices of healthcare personnel (HCP), considered the foundation of all efforts to reduce HAIs, has been a focus of many improvement efforts. Projects have focused on improving hand hygiene in intensive care units (ICUs), inpatient settings and emergency departments. They have addressed the behavior of nurses, physicians, and other HCP groups. Improvement efforts have also spanned the globe addressing infrastructure and societal challenges specific to improving hand hygiene in low- and middle-income countries (LMICs).^37–40^

Not long ago, poor hand hygiene behavior of HCWs was the norm, and device-associated infections were an expected complication of inpatient care. Thankfully, much has changed, improving in a stepwise fashion over time. Reports of HCP hand hygiene compliance was frequently very low, 40%–50%.^41^ Early projects were able to improve this to 66%–80% through promotion of alcohol-based hand rub use, education, and increased awareness.^42^ Subsequent projects have utilized different methods capable of achieving higher reliability, such as real-time feedback from peers or electronic reminders, that have resulted in sustained hand hygiene above 90%.^43–45^

### The ‘Big Four’: CLABSI, CAUTI, VAP, and SSI

Four HAIs are most commonly tracked, and these data are used to assess facilities’ patient safety and care quality: CLABSI, CAUTI, ventilator-associated pneumonia (VAP), and SSI. A common method to prevent these HAIs is control of a patient’s normal flora. Bacterial colonization on the skin of a patient may result in infection when allowed to colonize a catheter or contaminate a surgical wound. Likewise, excessive bacterial colonization of the mouth and oral secretions can increase the risk of VAP. Standardizing patient bathing, procedural skin preparation, and oral care have become important components of efforts to reduce device-associated infections and SSIs.^10,46,47^ Significant rate reduction in each HAI has been achieved through sharing of effective interventions from individual healthcare settings and multicenter collaborations that have utilized improvement science methodology to identify effective practices.^10,48–51^

### Prevention bundles

Determinants of HAIs are typically complex and multifactorial, and multimodal changes are required to reduce infections sustainably.^47^ Contrary to most research studies in which a single intervention is tested under very controlled circumstances, improvement science seeks to improve outcomes in real-world settings, often testing multiple interventions simultaneously. Implementing a group of interventions together, as a bundle, has become a global standard. The National Health and Safety Network reported mean ICU CLABSI rates during 2006–2008 of 1.3 to 5.5 per 1,000 line days.^52,53^ Development and implementation of insertion and maintenance bundles reduced rates to 1.4–2.3 CLABSIs per 1,000 line days in adults and children.^54,55^ Since that time, additional studies have refined these bundles and identified strategies to increase the reliability of bundle elements performance. Projects focusing on high-risk populations (premature neonates, oncology, intestinal failure, and cardiac patients) have implemented interventions addressing population-specific risks to expand CLABSI reduction to these groups as well.^48,56–58^ Once thought to be unachievable, rates below 1 CLABSI per 1,000 line days have become more commonplace.^56,59,60^

The use of more rigorous designs such as factorial design, which utilizes subgroups to simultaneously test multiple variables, can determine the relative impact of different interventions or the interaction of variables on the desired outcome. A multicenter collaborative to reduce CLABSIs in pediatric intensive care units (PICUs) utilized a factorial design and determined that the addition of chlorhexidine scrub and chlorhexidine impregnated sponges were not associated with reduced CLABSIs.^54^ Other project designs utilizing control units or cross-over designs can also be employed to increase confidence that changes made result in improved outcomes. A hospital-based project to improve healthcare worker hand hygiene sequentially tested interventions, including real-time reminders of healthcare workers forgetting to perform hand hygiene, on 2 different units. Improvement on each unit occurred only after the interventions were introduced.^61^

Although implementing evidence-based bundles can accelerate improvement, it can be difficult to determine which interventions are vital to improvement. From a practical standpoint, ensuring that each bundle component contributes directly to improved outcomes allows for efficient use of resources. However, retrospective studies of small and large databases have failed to identify which factors are necessary and sufficient to yield the desired outcome.^62,63^ Rather, reliability, the consistency of performing the bundle components at each opportunity, has been found to be an important part of any bundle.^10^ Improvement science methodology provides for measurement of both reliability and effect on outcome, and applying improvement science principles can ensure a clear association between tested interventions and improvement.

### Multicenter collaboratives

The urgency to prevent patient harm necessitates coordinated improvement efforts to increase the speed of learning, implementation, and spread of interventions to reduce HAIs. Multicenter collaboratives are an excellent platform to accelerate improvement through shared resources, learning and accountability. As previously discussed, they can also incorporate methodologic designs capable of more in-depth understanding of the impact of various interventions on improvement and the interaction of local context and culture on the effectiveness of interventions at different sites. Multicenter collaboratives have led to improved healthcare worker hand hygiene and reduced device-associated infections in the intensive care unit.^10,48,64,65^ For example, the Solutions for Patient Safety collaborative has reduced CLABSIs (13.7%), CAUTIs (56.6%) and SSIs (16.6%) since 2012.^11,66^ Another multicenter collaborative of 5 adult hospitals in Brazil reduced CLABSI, CAUTIs and VAPs.^51^ Regular communication and collaboration among collaborative members has been cited as a key factor for their success.^67^

Even though reaching zero HAIs may not be achievable, continued efforts to identify novel risk factors and to implement interventions to address them as well as application of improvement science to sustain improvements will continue to push the boundaries of preventable harm.

## Improvement science and antimicrobial stewardship

The threat of antimicrobial resistance, the need to limit unnecessary prescribing and the need for effective antimicrobial stewardship programs to improve clinical outcomes while reducing adverse effects is increasingly evident in inpatient, outpatient, and long-term care settings.^68^ Many of the factors affecting antimicrobial use are system-based and suitable for experimentation and optimization using improvement science methodology. Normative beliefs, observational learning experience, and assumed or vocalized patient expectations may push providers to prescribe antimicrobials when they are not indicated.^69,70^ A recent analysis from the United Kingdom reported that patients formed expectations of expectations, trying to read the prescribers’ intentions and reflect on the dependency between what prescribers and patients want.^71^ Reciprocal determinism is a model composed of 3 factors that influence behavior; the individual (including how they think or feel), their environment, and the behavior itself.^72^ For example, a parent of a sick child not wanting to miss work or with a false belief in antimicrobial effectiveness for viral illness may drive a demand for antibiotics despite a lack of indication.^73^ However, surprisingly, few antimicrobial stewardship programs are developed utilizing these improvement science concepts; therefore, they may not be as effective as they could be. Additionally, reports in the literature tend to be single-site limited engagement reports that are difficult to replicate and generalize to other contexts.

### Effective antimicrobial stewardship implementation

To be effective, antimicrobial stewardship implementation requires not only rapid knowledge transfer to the provider community for adoption of best practices but also the involvement of nurses, patients and families to accelerate organizational, programmatic, and cultural change. Many existing improvement science tools could be utilized to assess and implement programs. For example, individual barriers can be assessed using a knowledge, attitudes, and perceptions (KAP) survey for stakeholders or via the COM-B/Behavior Change Wheel (BCW) framework developed through review and synthesis of 19 existing behaving change frameworks.^74^ The COM-B/BCW framework provides a systematic method for identifying and organizing potential behaviors to a behavior change, selecting the barriers that are most likely to lead to the behavior change in each context and choosing evidence-based behavior change techniques most likely to be effective in overcoming targeted barriers. Prochaska and DiClemente’s transtheoretical model (TTM) is another framework consisting of 5 stages that conceptualize how people change their health behavior. This model has been broadly applied to infection prevention and control demonstrating the value in understanding motives among clinicians to adhere to standard precautions and success with stage-matched interventions.^75^

Organizational level strengths and barriers are also important to assess. Study outcomes can be evaluated using indicators to assess reach, effectiveness, adoption, implementation, and maintenance of the intervention (RE-AIM framework).^76^ A systematic review of antimicrobial stewardship in the ambulatory setting found that considering interactions between people and their workplace, the role of the physical environment and external pressures in antibiotic prescribing may be avenues for improvement.^77^

### Evidence for using improvement science in antimicrobial stewardship

To our knowledge, few studies have utilized improvement science in the implementation of antimicrobial stewardship programs in any setting. Meeker et al^78^ demonstrated in a randomized controlled trial in 5 outpatient primary care clinics, displaying poster-sized commitment letters (behavioral nudging that influences decision making) in patient examination rooms decreased inappropriate antibiotic prescribing for acute respiratory infections (ARIs). Quintos-Alagheband et al^79^ described developing an antimicrobial stewardship program with sustained reduction in antibiotic prescribing using a multifaceted quality improvement methodology that included a key driver diagram and rapid Plan–Do–Study–Act (PDSA) cycles.^79^ Brink et al^80^ incorporated results of a pre-implementation provider survey to implement antimicrobial stewardship process measures in a step-wise fashion in a diverse group of South African urban and rural private hospitals.

Yadav et al^81^ incorporated improvement science to address unnecessary antibiotic use for ARIs by existing antimicrobial stewardship programs in emergency departments and urgent care centers at 3 tertiary-care centers. A dynamic adaptation process was utilized to redesign the antimicrobial stewardship programs and interventions tested using a cluster-randomized comparative effectiveness clinical trial. Investigators adapted the CDC Core Elements for Outpatient Antimicrobial Stewardship by incorporating stakeholder interviews, validated provider surveys, workflow analyses and key personnel engagement.^82^ Data were collected on implementation process outcomes such as acceptability, fidelity, adoption, adaptation, and appropriateness. Results demonstrated that adaptation of the intervention components to the local setting followed by an implementation phase led to high acceptability and adoption of the intervention.

More studies in antimicrobial stewardship program implementation and evaluation through the lens of improvement science are needed to help demonstrate the practical utility of applying these theories and tools to further evolve the field of antimicrobial stewardship and tailor these interventions to a breadth of settings.

In conclusion, improvement science has been utilized to study and improve IP and antimicrobial stewardship efforts, but more work is necessary to control and eliminate HAIs and antimicrobial resistance. As healthcare contexts evolve, those involved in IP and antimicrobial stewardship efforts will be challenged to remain flexible and adaptive. Improvement science may be the answer for practitioners with limited resources and daunting challenges due to its framework and methodology that addresses systems, accounts for context, encourages rapid experimentation, and adjusts to achieve a goal. As the number and type of projects to reduce HAIs and promote antimicrobial stewardship expands, resources to support and coordinate this work will need to follow a similar trajectory. We encourage our colleagues conducting improvement science projects to share their experience, both positive and negative, with each other to promote common knowledge and collaboration to prevent harm. With the SQUIRE guidelines, precedent publications in both fields, and now the platform of ASHE, we look to move closer to our goals together.
